# Endemic *Acinetobacter baumannii* in a New York Hospital

**DOI:** 10.1371/journal.pone.0028566

**Published:** 2011-12-13

**Authors:** Scott A. Weisenberg, Audrey N. Schuetz, Elizabeth A. Alexander, Brain Eiss, Maryam Behta, Lisa Saiman, Davise H. Larone, Stephen G. Jenkins, Kyu Y. Rhee

**Affiliations:** 1 Department of Medicine, Weill Cornell Medical College, New York, New York, United States of America; 2 Department of Pathology, Weill Cornell Medical College, New York, New York, United States of America; 3 Department of Information Services, New York-Presbyterian Healthcare Medical Centers, New York, New York, United States of America; 4 Department of Pediatrics, Columbia University, New York, New York, United States of America; 5 Department of Infection Prevention and Control, New York-Presbyterian Hospital, New York, New York, United States of America; Los Angeles Biomedical Research Institute, United States of America

## Abstract

**Background:**

*Acinetobacter baumannii* is an increasingly multidrug-resistant (MDR) cause of hospital-acquired infections, often associated with limited therapeutic options. We investigated *A. baumannii* isolates at a New York hospital to characterize genetic relatedness.

**Methods:**

Thirty *A. baumannii* isolates from geographically-dispersed nursing units within the hospital were studied. Isolate relatedness was assessed by repetitive sequence polymerase chain reaction (rep-PCR). The presence and characteristics of integrons were assessed by PCR. Metabolomic profiles of a subset of a prevalent strain isolates and sporadic isolates were characterized and compared.

**Results:**

We detected a hospital-wide group of closely related carbapenem resistant MDR *A. baumannii* isolates. Compared with sporadic isolates, the prevalent strain isolates were more likely to be MDR (p = 0.001). Isolates from the prevalent strain carried a novel Class I integron sequence. Metabolomic profiles of selected prevalent strain isolates and sporadic isolates were similar.

**Conclusion:**

The *A. baumannii* population at our hospital represents a prevalent strain of related MDR isolates that contain a novel integron cassette. Prevalent strain and sporadic isolates did not segregate by metabolomic profiles. Further study of environmental, host, and bacterial factors associated with the persistence of prevalent endemic *A. baumannii* strains is needed to develop effective prevention strategies.

## Introduction

*Acinetobacter baumannii* is a nonfermentative, nonmotile, oxidase-negative, catalase positive gram-negative bacillus [1], and is an increasing cause of intensive care unit and hospital-associated infections [2]. Though the attributable mortality of *A. baumannii* infection continues to be debated [1], clinicians increasingly are confronted with infections with few or no antibiotic options. For this reason, *A. baumannii* has been identified as a particularly problematic pathogen by the Antimicrobial Availability Task Force of the Infectious Disease Society of America [3].

*A. baumannii* is now prevalent throughout New York City hospitals [4]. Like most hospitals in the area, NewYork-Presbyterian Hospital/Weill Cornell Medical College (NYPH/WCMC) has experienced an increase in *A. baumannii* infection and colonization during the last 10 years, though the relatedness of the isolates was largely unknown. We therefore studied the genetic relatedness of the hospital wide *A. baumannii* population at NYPH/WCMC during a period when the incidence and prevalence were stable. We subsequently compared the microbiologic, genetic, and metabolic characteristics of the prevalent endemic strain and sporadic isolates.

## Materials and Methods

### Isolate Selection

Institutional Review Board approval with waiver of informed consent was obtained. Isolates cultured at least 48 hours after admission between February and December 2008 were prospectively obtained from the Clinical Microbiology Laboratory, as were results of species identification and antimicrobial susceptibility testing. VITEK2 GN13 cards (bioMérieux Inc., Durham, NC) were used to identify members of the *A. calcoaceticus-A. baumannii* complex, and to assess antimicrobial susceptibility. In addition, Etests (AB bioMérieux, Durham, NC) were used to assess susceptibility to polymyxin B and tigecycline when requested by treating clinicians, or when the isolate was multidrug resistant. Multidrug-resistance (MDR) was defined as susceptibility to ≤1 tested antibiotic, excluding tigecycline and polymyxin B. No additional antimicrobial resistance testing was performed beyond what had been done for clinical purposes. Nursing unit and date of culture were obtained from the electronic medical record. Available *A. baumannii* isolates were intermittently collected from the microbiology laboratory during the study period. A total of 103 *A. baumannii* isolates from 50 patients were available, and only the first isolate per patient was used for this study. The total number of patients with an *A. baumannii* culture during the study period was 128. Isolates from 78 patients were unavailable due to sporadic collection of isolates. A convenience sample of 30 study isolates was selected from among the collected isolates to represent the largest number of intensive care units (ICUs) and wards in order to characterize the hospital wide *A. baumannii* population. The 19 excluded first isolates were from ICU patients whose ICU was already represented in the study database (12), non-ICU patients with contaminated plates (3), and from patients in hospital for less than 48 hours at the time of collection (4). The 12 excluded ICU isolates all contained the same integron pattern as the prevalent endemic isolates identified in the study. One of the 4 isolates collected in the first 48 hours was included (WC-31) since they had been transferred from an outside hospital and thus provided an opportunity to compare hospital strains. Only one isolate per patient was studied.

### Characterization of Isolates

All study isolates underwent Repetitive PCR (Rep-PCR) testing and Class I Integron analysis. After the initial analysis showed a prevalent endemic group of related isolates and additional sporadic isolates, we made the simplifying assumption that isolates with closely linked Rep-PCR results were of the same species and Multi Locus Sequence Typing (MLST) type. Thus, OXA-51-like gene amplification was done on all sporadic and the closest 5 prevalent endemic isolates, and MLST typing was done on a single prevalent endemic isolate. Metabolome analysis was performed on a subset of prevalent and endemic isolates. DNA templates and primers were prepared as described by Levasque et al. [5] with the exception that 3–5 colonies were dissolved in 100 µl of nuclease- free water, and selective antibiotics were not used. PCR amplification conditions for the 5′CS -3′CS integron analysis were as described by Martinez-Freijo et al. [6], with a two minute annealing time. A second PCR with non-overlapping primers was used to verify the integron contents of the prevalent endemic strain [7]. *A. baumannii*, OXA-51-like chromosomal genes were amplified from isolates WC 1–12 using previously described primers [8]. High Fidelity PCR Master mix was used for all reactions (Roche, Indianapolis, IN). PCR product nucleotide sequences were determined by Macrogen USA (Rockville, MD). Sequences were compared to known sequences using the Basic Local Alignment Search Tool (BLAST). MLST was performed using a selected isolate from the prevalent endemic strain and compared to the Pasteur MLST site (http://www.pasteur.fr/recherche/genopole/PF8/mlst/Abaumannii.html).

### Repetitive-PCR typing

DNA was extracted using the UltraClean™ microbial DNA isolation kit (MO BIO Labs, Inc., Solana Beach, CA). Rep-PCR was performed using the DiversiLab system (bioMérieux Inc., Durham, NC). The model 2100 bioanalyzer and LabChip reagents (Agilent Technologies Inc., Palo Alto, CA) were used for DNA separation. The DiversiLab system created dendrograms, scatterplots, electropherograms, and virtual gel images for data analysis. Similarity criteria were followed as per Saeed [9].

### Metabolome Analysis

Differences in the metabolic properties of selected isolates were studied by examining individual metabolites of interest and global metabolic profiles. Biological replicates of *A. baumannii* isolates were cultivated in phosphate-M9 minimal media [10] and grown for metabolomic profiling on phosphate-M9 agar [11]. Bacterial isolates were harvested during mid-logarithmic phase of growth, and metabolites were isolated as described previously [12]. Variations in biomass were normalized for all isolates by analysis of residual protein content [13]. An Agilent Accurate Mass 6220 time-of-flight mass spectrometer coupled to an Agilent 1200 liquid chromatography system was used as described previously [12]. Data were collected in centroid mode in 2 GHz (extended dynamic range) mode. Detected masses were deemed identified metabolites on the basis of unique accurate mass retention times which confirmed to the expected metabolites' mass and that of accompanying isotopomers [14]. Deconvolution of mass spectra was performed using the Agilent MassHunter Qualitative Analysis molecular feature extraction algorithm which identifies individual metabolites using chromatographically covariant ion families which conform to specific empirical formulae [14]. The normalized ion counts (in triplicate) of 3 prevalent endemic strain isolates were compared to the ion counts of 3 sporadic isolates (in triplicate), and compared using Stata version 10.0 by Wilcoxon Rank-Sum test. Additional statistical analyses used GeneSpring MS software to perform principle components analysis (PCA) and hierarchical clustering analysis (HCA).

## Results

### Study Isolates and Antimicrobial Susceptibility

Thirty isolates from unique patients were studied (Table 1). The study isolates represented 24.2% of the 128 *A. baumannii* isolates cultured from unique patients during that period. Patient locations at the time of culture were geographically widespread, including seven ICUs (5 floors) and seven non-ICU nursing units (5 floors) over a 9 month period. An additional isolate (WC-31), cultured on the first day of hospitalization from a patient transferred to NYPH/WCMC from another hospital in Brooklyn in New York City, was also included in the rep-PCR and integron analyses.

**Table 1:** Study Isolates.

*WC*	*PT*	*Date*	*FLOOR*	*A/S*	*Carb*	*Cef*	*Gent*	*Amik*	*S/T*	*LEVO*	*PM*	*Tig*	*SOURCE*
1	68F	9/5	A ICU	S	S	S	S	S	R	S			RESP
2	48M	2/29	A ICU	R	R	R	R	R	R	I	0.8	4	RESP
3	12F	9/26	B ICU	S	S	S	S	S	S	S			RESP
4	18F	8/3	B ICU	S	S	S	S	S	S	S		0.5	RESP
5	58M	10/8	C ICU	S	S	I	S	R	R	I			RESP
6	40F	7/30	B WARD	S	S	S	S	S	S	S			URINE
7	79F	8/24	D ICU	S	S	S	S	S	S	S			RESP
8	76M	9/22	E ICU	S	R	R	R	R	R	R	1	12	RESP
9	76F	10/26	C ICU	R	R	R	R	I	R	I	0.75	4	RESP
10	71M	11/7	D WARD	R	R	R	R	R	R	R	1	12.5	BLOOD
11	3F	8/8	B ICU	R	R	R	R	R	R	I	1.5	3	RESP
12	6M	9/15	A ICU	R	R	R	R	S	R	I	1.5	3	URINE
13	78F	7/13	D WARD	S	R	R	R	R	S	S	1	3	URINE
14	46F	6/24	D ICU	R	R	R	R	R	R	R	4	2	BLOOD
15	67M	9/20	E ICU	R	R	R	R	I	R	R	1	2	RESP
16	67M	9/2	A WARD	R	R	R	S	I	R	I	1.5	3	URINE
17	34M	11/23	A ICU	R	R	R	R	R	R	R	2	4	RESP
18	71F	10/26	A ICU	R	R	R	R	I	R	I	0.5	2	BLOOD
19	62M	12/6	D ICU	R	R	R	R	R	R	R	0.5	6	RESP
20	87F	11/5	F WARD	R	R	R	R	R	R	R	0.5	8	BLOOD
21	78M	11/30	E WARD	R	R	R	R	R	R	R	2	2	BLOOD
22	65F	9/20	E ICU	R	R	R	R	S	R	R	0.75	2	BLOOD
23	43M	8/18	A ICU	R	R	R	R	S	R	I	32	4	RESP
24	76M	8/10	E ICU	R	R	R	R	R	R	I	0.5	2	BLOOD
25	20M	9/21	A ICU	R	R	I	R	I	R	I	2	2	RESP
26	65F	11/9	C ICU	R	R	R	R	R	R	I	2	3	RESP
27	71M	9/6	D WARD	S	I	I	S	S	R	R		2	URINE
28	45F	9/16	C ICU	S	S	R	R	S	R	R		2	RESP
29	50M	3/11	A ICU	R	R	R	R	R	R	R	0.5	3	URINE
30	61F	8/26	D ICU	R	R	R	R	R	R	R	1.5	2	BLOOD
31	78M	11/10	A WARD	S	R	I	R	S	R	R	0.5	2	URINE

Study isolates (plus WC-31, which was isolated from a patient on Hospital Day 1 after transfer from an outside hospital). **Legend WC** = Weill Cornell; **PT** = Patient age and gender; **Date:** All dates are from 2008; **Floor:** Each letter represents a different hospital floor; Antimicrobial susceptibility to the various agents was determined following CLSI guidelines, or the Mean Inhibitory Concentration is reported directly for polymyxin B and tigecycline (µg/mL): A/S: ampicillin-sulbactam, Carb: imipenem or meropenem; Cef: cefepime; Gent; gentamicin; Amik: amikacin; S/T: sulfamethoxazole-trimethoprim; Levo: levofloxacin; PM: polymyxin B; Tig: tigecycline; Source: Resp = respiratory. Susceptibility interpretation based on 2008 CLSI cutoff points.

The majority (70%) of the isolates in this study were multidrug-resistant. The proportion of isolates susceptible to tested antimicrobial agents included ampicillin-sulbactam (33%), imipenem or meropenem (23%), cefepime (17%), gentamicin (27%), tobramycin (33%), amikacin (33%), trimethoprim-sulfamethoxazole (17%), and levofloxacin (20%). In contrast, 20 of the 22 (91%) isolates tested were susceptible to polymyxin B (MIC≤2 µg/mL), though the MICs for eight were between 1.5 and 2 µg/mL. Tigecycline MICS were ≤2 µg/mL for 11 of the 25 (44%) isolates tested. The chromosomal *A. baumannii* gene *bla*-OXA-51-like carbapenemase gene [8] could not be amplified from four isolates (WC 1–4), suggesting that they belonged to other *A. calcoaceticus-A. baumannii* complex species. Pending further identification, these isolates are considered “*A. baumannii*” in this study.

### Molecular Epidemiology

**Figure 1:**
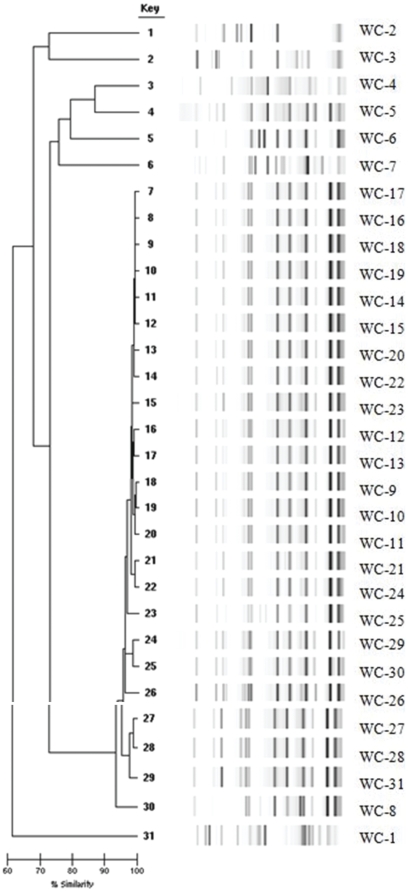
Rep-PCR Analysis of Study Isolates. Isolates with more than 90% similarity were considered related. Study isolates (WC 1–30) are shown, plus one isolate (WC-31) isolated on hospital day 1 after transfer from an outside hospital. Results show the majority of the study isolates are closely related.

Rep-PCR testing showed that 23 of 30 (77%) study isolates were closely related with similarity scores over 90% (Figure 1). This prevalent endemic strain of 23 isolates, hereafter referred to as the “prevalent strain”, was geographically widespread and cultured from patients in 7 ICUs and 6 non-ICU nursing units. The isolate obtained from a patient transferred from a Brooklyn hospital (WC-31), was highly similar to the prevalent strain. MLST of a one prevalent strain isolate (WC-9) was consistent with ST-2, a member of Worldwide Clone II [15].

Seven additional isolates were not closely related to the prevalent group, or to each other, hereafter referred to as “sporadic isolates”. One of the seven (14.3%) sporadic isolates was MDR, compared with 20 of 23 (87%) prevalent strain isolates. Prevalent strain isolates were thus more likely to be MDR than the sporadic isolates (two tail Fisher's Exact p = 0.001).

### Integron Assessment of Prevalent A. baumannii

**Figure 2:**
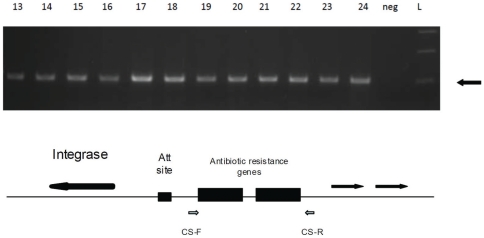
Integron Content in Select Study Isolates. Integron PCR gene products of WC 13–24 are shown. The block arrow marks the 550 base pair PCR product of the prevalent strain *A. baumannii*. The water control is labeled “neg.” The cartoon shows the layout of a typical Class I integron. The PCR primers are presented by CS-F and CS-R.

Amplification of the integron variable region showed that all prevalent strain isolates contained a common 550 base pair product (GenBank # JF309278, Figure 2). In contrast, the sporadic isolates contained products of varying sizes (data not shown), though two also contained an identically sequenced 550 base pair product. BLAST sequence analysis of the 550 base pair integron variable region showed 100% homology with a non-integron-associated *A. baumannii*-associated sequence, which included a portion of a glucose-sorbosone dehydrogenase encoding gene (gb|CP000863.1). The same PCR sequence was amplified using alternative, non-overlapping Class I integron primers [7], confirming that this sequence was integron-associated in the study isolates.

### Metabolome Analysis

**Figure 3:**
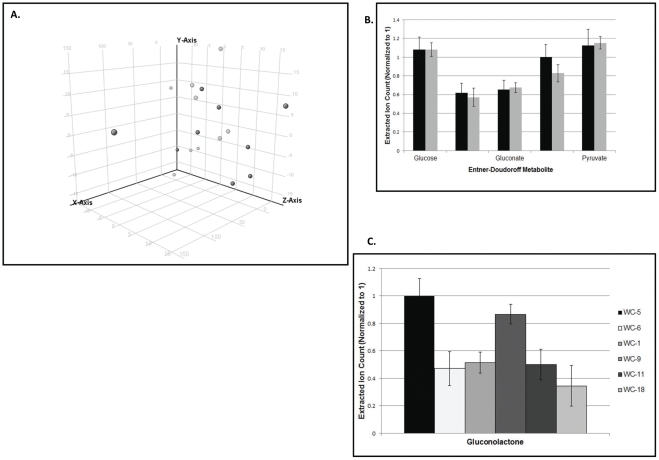
Metabolomic Analysis of Prevalent Endemic and Sporadic *A. baumannii* Isolates. (**A**) Principal components analysis of prevalent endemic (black circles) and sporadic (gray circles) isolates (in which the metabolomic profile of each isolate is represented as a single point on a 3-D axis) shows significant overap, consistent with similar metabolic profiles. (**B**) Targeted analysis of intermediates of the Entner-Doudoroff (ED) pathway shows no change in abundance of ED metabolites in sporadic (gray) vs prevalent endemic (black) isolates. (**C**) Abundance of gluconolactone (a representative intermediate in the ED pathway) did not differ significantly between sporadic isolates (WC-1, WC-5, and WC-6) and prevalent endemic isolates (WC-9, WC-5, and WC-6).

Metabolic characteristics of both prevalent strain and sporadic isolates were compared by global metabolomic analysis and targeted analysis of individual metabolites. Biological replicates of three prevalent strain isolates (WC-9, WC-11, and WC-18) and 3 sporadic isolates (WC 1, WC-5, and WC-6) were selected and cultured using glucose as the sole carbon source. *Acinetobacter* species are known to metabolize glucose through the Entner-Doudoroff pathway [16]. The prevalent strain isolates and sporadic isolates did not differ in metabolites of glucose oxidation or the Entner-Doudoroff pathway (Figure 3). Finally, we compared the global metabolome profiles of prevalent strain isolates and sporadic isolates using Principle Components Analysis and Hierarchical Clustering Analysis. No significant differences in the global metabolite profiles of prevalent strain isolates and sporadic isolates were noted (Figure 3).

## Discussion

*A. baumannii* is now endemic throughout NYC [4]. A limited number of circulating *A. baumannii* strains, some from multiple institutions and other from single sites, have been noted in previous studies [17]–[20]. The findings from our study, add to existing knowledge by showing the geographically wide distribution of a prevalent strain within one hospital during a period when no hospital-wide outbreak was suspected. The prevalent strain is a member of the expanding Worldwide Clone II, formerly known as European Clone II [15]. Persistence in hospital environments over prolonged periods appears to be a characteristic of successful *A. baumannii* strains [21], [22].

The prevalent *A. baumannii* strain contained a unique Class I integron sequence. This sequence was also amplified from two sporadic isolates, suggesting horizontal transfer. Integrons have been associated with prevalent *A. baumannii* strains [23], increased resistance to antibiotics [24], and persistence in hospital environments [25]. The significance of the partial glucose sorbosone dehydrogenase gene sequence encoded in the prevalent integron remains to be elucidated.

It is unclear why some isolates of *A. baumannii* become epidemic or endemic, and others remain sporadic. The virulence and persistence mechanisms of *A. baumannii* are only beginning to become understood [26], though significant genomic differences in *A. baumannii* isolates that are associated with human, environmental, or lice host environments have been noted [27]. Epidemic *A. baumannii* are able to survive on dry surfaces for prolonged periods in hospital environments, but do not persist longer than sporadic *A. baumannii* [28]. Antibiotic resistance itself is associated with increased persistence of some *A. baumannii* strains compared with more susceptible strains [29], though the relative contribution of antibiotic resistance and other unspecified virulence factors remains unknown. We found no significant metabolic differences between a limited number of tested sporadic isolates and prevalent strain isolates. While varying environmental conditions may lead to differences in isolates [30], our results are consistent with a primary role for antibiotic resistance itself as a marker for *A. baumannii* strains with increased potential to persist in hospitals. Furthermore, in contrast to previous metabolomic analysis of methicillin-susceptible and -resistant *Staphylococcus aureus*, as well as vancomycin-susceptible and -resistant *Enterococcus faecium*, the metabolic profiles of the isolates did not segregate by antibiotic resistance type [12]. This raises the question of whether antibiotic resistance in *A. baumannii* requires less metabolomic adaptation (or “cost”) compared with these gram-positive organisms. Metabolomic characteristics of *A. baumannii* have been investigated [31], but strain to stain metabolomic variability had not been described to our knowledge. Further study is needed to clarify the metabolic, proteomic, and genomic differences associated with prevalent and sporadic MDR *A. baumannii*.

The study has several limitations. We studied a non-random convenience sample of isolates selected to represent hospital-wide locations, which raises the potential of selection bias. Though Rep-PCR was not done on the available but excluded isolates, antibiogram and integron analysis suggest most excluded isolates belonged to the prevalent strain (data not shown). Only a subset of the total *A. baumannii* isolates from the hospital were collected over the study period, which may introduce additional bias if the collected isolates differed in any way from the hospital wide isolates. However, the antibiotic susceptibility profile of the study isolates was similar to that of isolates from both ICU and non-ICU nursing units, suggesting that study isolates were representative of the hospital-wide *A. baumannii* strains. Our study period was only nine months and, thus, it is possible that we detected an unrecognized outbreak, rather than an endemic period, although the incidence and prevalence of *A. baumannii* had been stable for three years at the time of the study (data not shown). It is also possible that a longer sampling period would have uncovered additional prevalent strains of *A. baumannii*, such as those detected elsewhere in NYC [18]. Ongoing molecular epidemiologic surveillance of cultured isolates may further clarify the long term dynamics of the prevalent endemic *A. baumannii* strain within the hospital. Finally, isolates closely related by Rep-PCR may evolve clinically important differences, such as the differences in antimicrobial susceptibility testing results seen within the prevalent strain. Unrelated isolates may exchange genetic information and potentially share traits, highlighted by the finding of the prevalent strain integron in two of the sporadic isolates.

This study characterized the *A. baumannii* isolates from a NYC hospital. A previously unsuspected hospital-wide prevalent strain of multidrug-resistant *A. baumannii* was uncovered. The prevalent strain contained a novel integron cassette, though the functional significance of this finding requires further study. Metabolomic analysis did not segregate the prevalent strain from sporadic isolates. Identification of environmental, patient, and bacteria-specific features associated with the development of persistent *A. baumannii* strains will aid efforts to combat this emerging pathogen.
