# Correction: Endemic *Acinetobacter baumannii* in a New York Hospital

**DOI:** 10.1371/annotation/22d2ce95-f6c5-46fa-9e32-0d0fd21e20df

**Published:** 2012-10-24

**Authors:** Scott A. Weisenberg, Audrey N. Schuetz, Elizabeth A. Alexander, Brain Eiss, Maryam Behta, Lisa Saiman, Davise H. Larone, Stephen G. Jenkins, Kyu Y. Rhee

The third author's name was spelled incorrectly. The correct name is: Elizabeth L. Alexander.

The correct citation is: Weisenberg SA, Schuetz AN, Alexander EL, Eiss B, Behta M, et al. (2011) Endemic *Acinetobacter baumannii* in a New York Hospital. PLoS ONE 6(12): e28566. doi:10.1371/journal.pone.0028566 

